# Un cas rare de luxation sous-talienne pure

**DOI:** 10.11604/pamj.2015.20.442.6749

**Published:** 2015-04-30

**Authors:** Soufiane Guelzim, Mustapha Mahfoud

**Affiliations:** 1 Soufiane Guelzim, Service de Chirurgie Orthopédique et Traumatologie, CHU Ibn Sina, Rabat, Maroc

**Keywords:** Luxation, sous-talienne, articulation talo-crurale, dislocation, subtalar, ankle joint

## Image en medicine

La luxation sous-talienne est une perte de rapports anatomiques entre l'astragale, calcanéum et scaphoïde. C'est une lésion rare, elle représente 1% de toutes les luxations observées en traumatologie. Nous rapportons le cas d'une patiente de 40 ans, sans antécédents, ayant subi un traumatisme de son pied droit lors de la réception d'un saut d'un lieu élevé d'un mètre (mécanisme en inversion du pied bloqué en équin). Le diagnostic était évident cliniquement devant la déformation douloureuse du pied avec impotence fonctionnelle: le talon est déplacé médialement par rapport à la jambe, le pied étant en inversion, flexion plantaire et adduction avec raccourcissement du bord médial du pied et tension cutanée. Il n'y avait pas d'ouverture cutanée et l'examen vasculo-nerveux était normal. Le bilan radiographique initial avait objectivé cette luxation sous-talienne interne pure sans fractures associées. Le scanner du pied et de la cheville a confirmé ces lésions. La réduction orthopédique urgente par manoeuvres externes, a été faite dans l'heure suivant le traumatisme au bloc opératoire sous rachianesthésie et sous contrôle fluoroscopique. Après réduction, l'articulation talo-crurale était stable et le contrôle radiologique a objectivé une bonne congruence articulaire. Une contention complémentaire par botte plâtrée a été réalisée et maintenue pendant 6 semaines sans appui, puis la rééducation a été entreprise. L’évolution a été favorable, le résultat fonctionnel est très bon après un recul de 24 mois.

**Figure 1:**
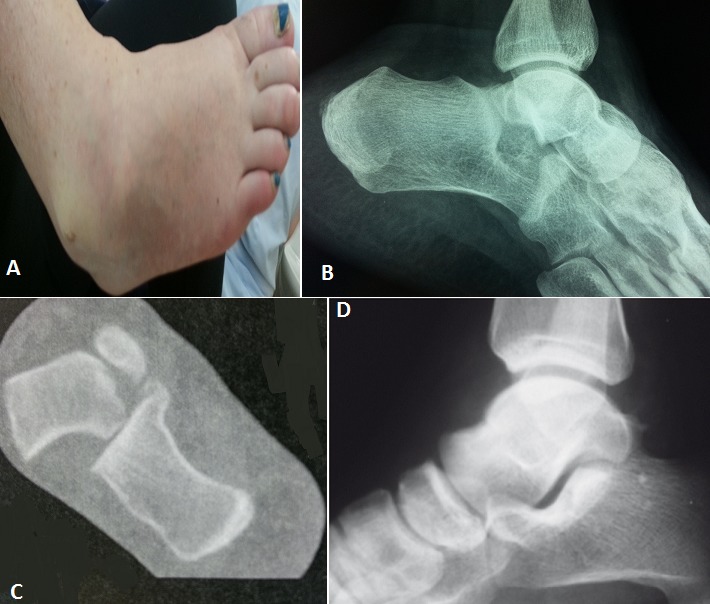
(A) image Clinique; (B) radiographie standard de profil de la cheville et du médio-pied; (C) scanner de la cheville et du pied confirmant la lésion; (D) contrôle radiographique de la réduction orthopédique avec une bonne congruence articulaire

